# Identification and Chemical Control of Stem Canker Pathogen of *Idesia polycarpa*

**DOI:** 10.3390/plants14091393

**Published:** 2025-05-05

**Authors:** Jian Feng, Qiupeng Yuan, Xuzhong Chen, Lisha Fang, Tao Zhang, Zhen Liu, Yanmei Wang, Xiaodong Geng, Qifei Cai, Zhi Li

**Affiliations:** 1 College of Forestry, Henan Agricultural University, Zhengzhou 450046, China; 2 National Forestry and Grassland Administration Key Laboratory for Central Plains Forest Resources Cultivation, Zhengzhou 450046, China; 3 Henan Province Engineering Technology Research Center for Idesia, Zhengzhou 450046, China; 4 Key Laboratory for Bio-Resource and Eco-Environment of Ministry of Education, College of Life Sciences, Sichuan University, Chengdu 610065, China; 5 Zhongyuan Environmental Protection Development Co., Ltd., Zhengzhou 450046, China

**Keywords:** *Idesia polycarpa*, stem canker, *Botryosphaeria dothidea*, wood pathogens, fungicide

## Abstract

*Idesia polycarpa* is an important woody oilseed tree crucial for ensuring China’s grain and oil security. The expansion of *I. polycarpa* plantations has been accompanied by an increase in pests and diseases, with canker disease recently observed in two forests in Henan Province. Field surveys revealed a disease incidence of 70.12% among 328 surveyed trees, indicating a substantial threat to plantation health. The most virulent pathogen, strain SQ5, was identified as *Botryosphaeria dothidea* through molecular sequencing and morphological analyses. Strain SQ5 showed an optimum growth temperature of 25 °C and a mycelial lethal temperature of 60 °C. The pathogen thrives in acidic conditions and is promoted by light, with the ability to utilize various carbon and nitrogen sources. In vitro toxicity assessments identified four effective fungicides: 70% thiophanate-methyl (EC50 = 0.0169 µg/mL), 43% tebuconazole (EC50 = 0.0219 µg/mL), 20% octylamine acetate (EC50 = 0.0271 µg/mL), and 40% difenoconazole (EC50 = 0.0954 µg/mL). Field trials demonstrated that 43% tebuconazole (average efficacy = 35.29%) and 40% difenoconazole (average efficacy = 23.53%) exhibited superior control of *I. polycarpa* canker. This study represents the first systematic analysis of *I. polycarpa* canker and its control measures, laying a foundation for further research and field management strategies. Given the significance of *I. polycarpa* in Chinese forestry, this underscores the need for effective management strategies to sustain its productivity and mitigate risks associated with expanding plantations.

## 1. Introduction

Canker is a prevalent plant disease with widespread detrimental effects, impacting broad-leaved trees, coniferous trees, and herbaceous plants [1,2,3]. In particular, forest cankers cause localized necrosis in the trunk and branch cortex [4]. In mild cases, the disease disrupts the transport of nutrients and water within the tree, thereby impeding its growth and development. Without intervention, the disease progresses, significantly inhibiting growth and eventually leading to tree mortality. Notably, in 1994, 3000 beech trees succumbed to canker in the Great Smoky Mountains National Park, USA [5]. In 2015, *Populus tremuloides* across six ecological zones in Alaska experienced a 70% mortality rate due to canker disease [6]. Since 2018, *Pinus armandii* in Jingbian Village, Zhouqu County, China, has been affected by canker disease, with incidence rates increasing annually and reaching up to 80% in local plots [7]. Forest canker has emerged as a significant secondary forest pest in China, posing a threat to the forestry industry’s sustainable development [8].

*Idesia polycarpa*, a dioecious plant from the genus *Idesia* within the family Flacourtiaceae, stands out as an ideal tree species for ecological enhancement due to its high adaptability, drought and barrenness tolerance, and minimal climatic requirements [9]. Additionally, it serves as a high-quality woody oil species, with oil content reaching 37.01% in the fruit pulp, 21.49% in the seeds, and 30.00% in the dried fruits [10]. These values surpass those of competitors such as *Camellia oleifera* and *Olea europaea* in the current market [11]. *I. polycarpa* oil is distinguished by its high quality and richness in unsaturated fatty acids, particularly linoleic acid, which constitutes an impressive 73.00% [12], making it a premium edible oil. Currently, *I. polycarpa* has been included in the national reserve forest initiative for superior industrial tree species, and its oil is classified as a general food product by the state [13]. The *I. polycarpa* industry has entered a phase of rapid and efficient growth, with promising market prospects. In recent years, the state has consistently increased support for the woody oilseed industry, implementing a series of policies [14]. Large-scale cultivation has been carried out across various regions, with *I. polycarpa* plantations covering 3300 km^2^ in Hubei Province, over 66.67 km^2^ in Sichuan Province, more than 13,333 km^2^ in Shaotong City, Yunnan Province, and 1000 km^2^ in Luoyang City, Henan Province, as of spring 2021. With growing recognition of *I. polycarpa*’s value, its cultivation area is anticipated to expand further.

It is generally believed that *I. polycarpa* has strong adaptability and resistance to diseases and pests. However, with the development of the *I. polycarpa* industry and the gradual expansion of artificial plantation areas, various diseases have emerged under the influence of environmental and human factors, seriously affecting the economic value of *I. polycarpa* and hindering the industry’s healthy development. In a study, Zhou et al. [15] identified multiple diseases across seven *I. polycarpa* plantations in Sichuan Province, with plaster disease and anthracnose being the most common and damaging. Zhu [16] confirmed the presence of six types of diseases, including damping-off, anthracnose, and gray patch disease, in major *I. polycarpa* cultivation areas in Zhejiang Province. Notably, a new disease symptom was observed in two *I. polycarpa* forests in Henan Province: circular brown spots with water-soaked edges on tree stems, resembling canker symptoms previously reported in other tree species. Currently, there are no systematic reports on this disease affecting *I. polycarpa*.

Given this context, the objective of this study was to determine the incidence pattern of canker in *I. polycarpa* through field surveys. Subsequently, the aim was to identify the pathogen causing *I. polycarpa* canker through tissue culture, morphological observation, and molecular techniques, providing a theoretical basis for its prevention and control. Additionally, a pharmaceutical test was conducted to screen effective fungicides, offering a scientific reference for the field control of *I. polycarpa* canker.

## 2. Results

### 2.1. Symptoms of I. polycarpa Canker

**Figure 1:**
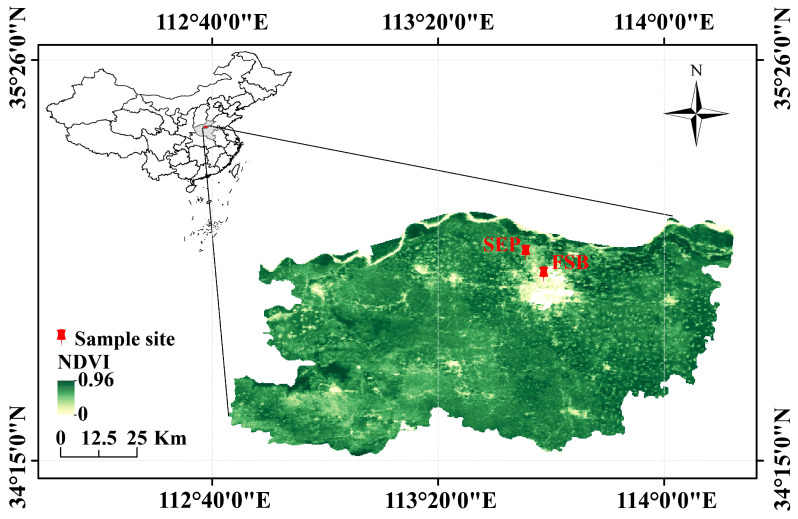
The spatial distribution of sampling sites in Zhengzhou, Henan Province, China. NDVI values were obtained from the MOD13Q1 product.

**Figure 2:**
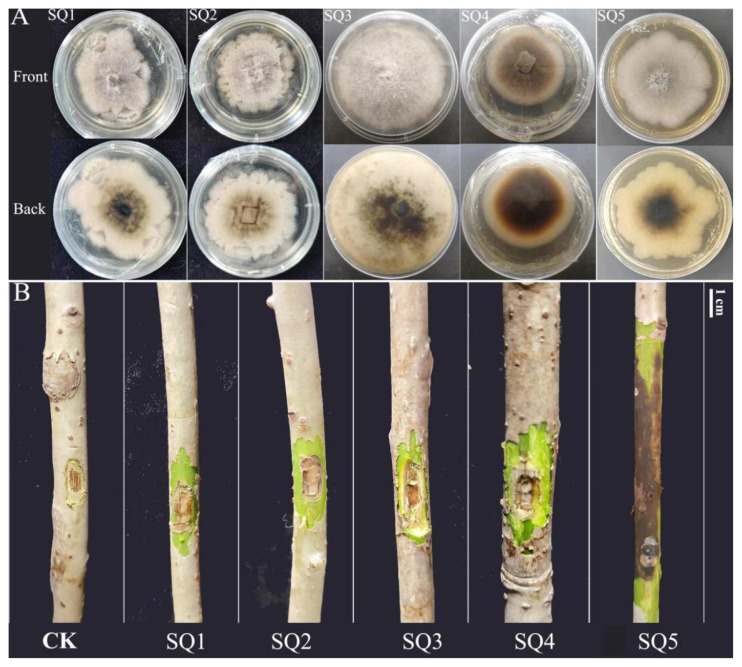
Pathogen isolation and inoculation. (**A**) Five isolated fungi of *Botryosphaeria dothidea*. (**B**) Inoculation of *I. polycarpa* seedlings.

Between 2020 and 2021, field surveys were conducted on *I. polycarpa* at two sites (Figure 1), based on typical canker disease symptoms, including trunk-localized lesions, circular brown spots, a water-soaked appearance, and internal tissue decay (Figure 2). 

**Figure 3:**
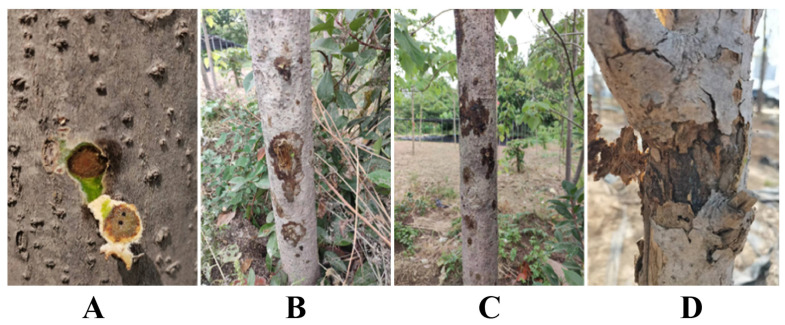
Field symptoms of *Idesia. polycarpa* canker disease. (**A**–**D**) depict a progressive aggravation of *I. polycarpa* canker symptoms observed in the field.

A total of 328 trees were assessed, of which approximately 230 showed typical canker symptoms, and 17 symptomatic individuals died during the observation period. Disease incidence was 70.12%, calculated as the proportion of symptomatic trees among all surveyed individuals. These results indicate that canker disease poses a significant threat to the growth and survival of *I. polycarpa*. The disease typically initiates in mid to late March and persists until mid to late September. Considering the climate change patterns in Zhengzhou City (Figure S1), it can be concluded that its onset is concentrated in the hot and rainy seasons. Canker disease predominantly affects trees over 5 years old, with susceptible parts concentrated in the trunk. As the disease progresses, parts of the branches and trunk will also appear as cankers. In the early stages, a sub-circular brown spot appears on the affected stem area, releasing brown fluid upon pressing, indicating internal decay (Figure 3A). Progressing further, the spots extend along the trunk into the xylem, forming patches that release significant amounts of brown water (Figure 3B,C). In later stages, the spots become sunken and the epidermis cracks, causing branches or the entire plant to die (Figure 3D). Additionally, we noted that on sunny days following rain, the severity of *I. polycarpa* canker increases, as observed after several days of intense heat. This pattern aligns with the overall morbidity trend observed in *I. polycarpa*.

### 2.2. Isolation of Pathogen and Determination of Pathogenicity

A total of 27 strains were isolated from the boundary between diseased and healthy tissues of *I. polycarpa*. Based on literature reviews, non-pathogenic strains were excluded. Preliminary identification, considering colony morphology and disease symptoms observed in the field, suggested that the pathogen belongs to the genus *Botryosphaeria*. Five representative strains (SQ1, SQ2, SQ3, SQ4, and SQ5) were selected for pathogenicity assays (Figure 2A). Branches inoculated with agar blocks showed no symptoms, whereas inoculation with these strains induced disease symptoms, including discoloration and depressed lesions at the inoculation sites (Figure 2B). Notably, branches inoculated with strain SQ5 exhibited significantly larger depressions compared to other strains, with some seedlings showing leaf wilting and abscission. Re-isolated colonies from infected tissues displayed morphological characteristics (color, shape, hyphal texture) consistent with the original strain, thus fulfilling Koch’s postulates. Strain SQ5 was therefore identified as the most virulent pathogen.

### 2.3. Morphological and Cultural Characteristics

**Figure 4:**
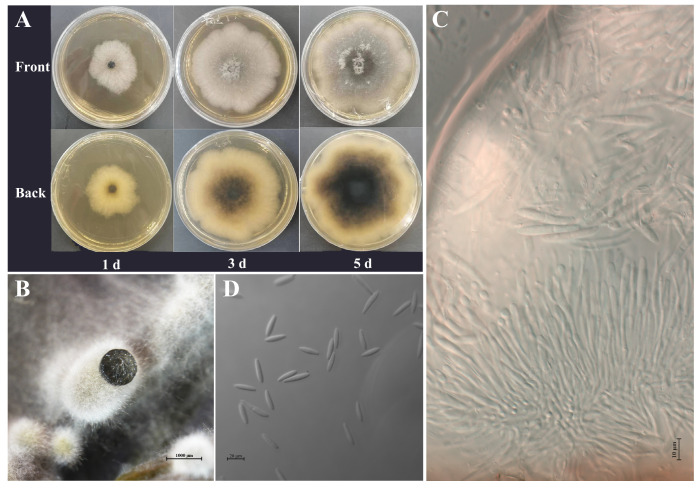
Morphological characteristics of *B. dothidea*. (**A**) Changes in colony morphology. (**B**) Section views of pycnidia. (**C**) Conidiophore. (**D**) Conidia.

The SQ5 pathogen exhibits rapid growth, covering the entire plate within 5 to 6 days (Figure 4A,B). The colony is suborbicular with well-defined edges and dense mycelium. In the early stages of colony development, the mycelium appears white. By the third day, dark green or black pigmentation begins to emerge approximately 6.00 mm from the center of the colony. Simultaneously, aerial mycelium develops within the colony, characterized by thick, cotton-like growth that can stand erect. By the fifth day of incubation, the entire colony adopts a dark-green color. The pathogen was cultured in a spore-producing medium, leading to conidiophore production after approximately 14 days. Through light microscopy, the sporophore was observed to be colorless, rod-shaped, and producing conidia at its apex (Figure 4C). The conidia were spindle-shaped or fusiform, smooth, and non-septate (Figure 4D). Measurements of 30 conidia revealed that their size ranged from 23.678 to 30.332 μm in length and 5.132 to 6.795 μm in width, with an average size of 26.185 μm × 6.157 μm. These findings are consistent with the morphological characteristics of *B. dothidea* reported by Zheng et al. [17], He et al. [18], and Zhang et al. [19].

### 2.4. Molecular Identification and Phylogenetic Analyses

To validate the morphological identification, we amplified ITS, β-*tubulin*, and *tef1*-α gene fragments from the SQ5 isolate. PCR amplification of the ITS region, β-*tubulin*, and *tef1*-α produced fragments of 523 bp, 432 bp, and 259 bp, respectively. The generated ITS, β-tubulin, and tef1-α sequences were submitted to GenBank (Table 1). 

**Figure 5:**
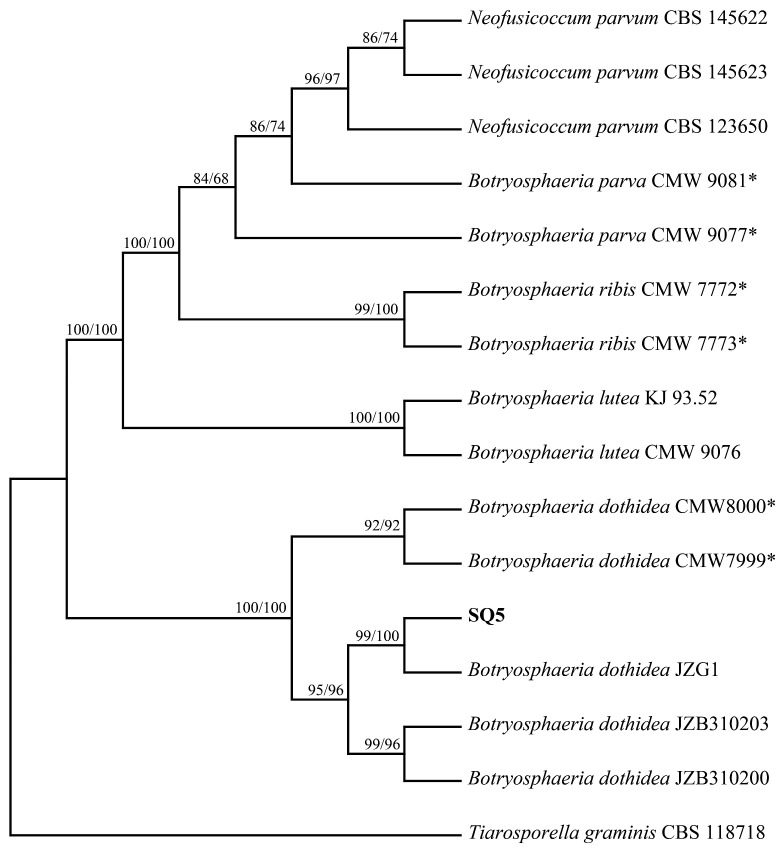
One of 27 equally parsimonious trees generated from maximum-parsimony analysis of the three-gene (ITS + tef1-α + β-tubulin) dataset. Numbers represent bootstrap values (MP/ML) which >70%. Isolates in bold were generated in this study. The ex-type strain is marked with *. *Tiarosporella graminis* was used to out-group.

Preliminary alignment of these sequences with the NCBI database revealed that the SQ5 strain showed high similarity to *Botryosphaeria*. Phylogenetic analysis (Figure 5), based on the three-locus dataset (ITS + *tef1*-α + β-*tubulin*) and including 16 taxa, resulted in a dataset of 1326 characters. Of these, 156 characters were parsimony informative, 108 characters were parsimony uninformative, and 1062 characters were constant. A total of 27 trees were retained for the MP analysis (tree length = 313, CI = 0.939, RI = 0.972, RC = 0.913). Maximum Likelihood (ML) and Maximum Parsimony (MP) analyses based on the combined sequences revealed that SQ5 clustered with the previously reported *B. dothidea* JZG1 with 99% bootstrap support, clearly distinguishing it from other species of *Botryosphaeria*. Therefore, SQ5 was identified as *B. dothidea*.

### 2.5. Biological Characteristics

**Figure 6:**
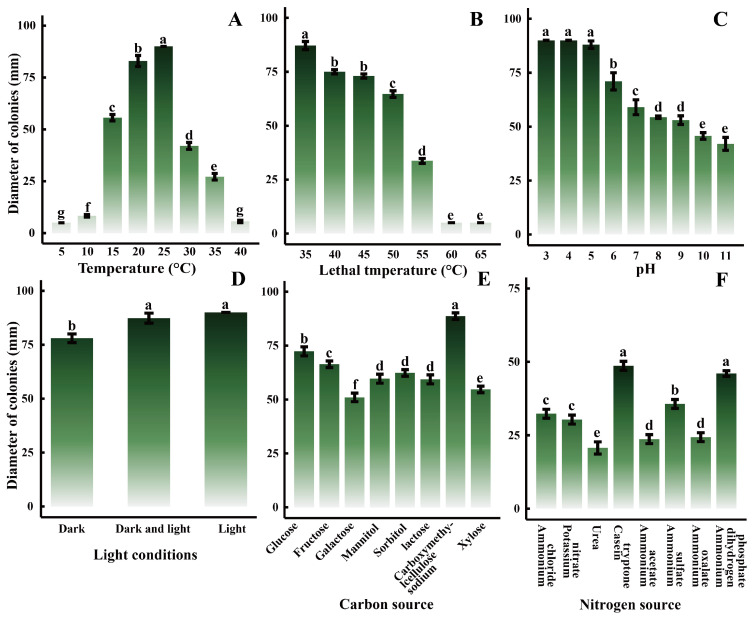
Effect of different environmental conditions on the growth of strain SQ5. (**A**) Temperature. (**B**) Lethal temperature. (**C**) pH; (**D**) Light conditions. (**E**) Carbon source. (**F**) Nitrogen source. Different lowercase letters represent significant differences (*p* < 0.05).

The growth of pathogens is closely linked to environmental conditions, prompting an investigation into their growth under various settings. The results indicated that the pathogen could thrive within a temperature range of 10–35 °C (Figure 6A), with significant differences observed (*p* < 0.05). After 5 days of incubation, the maximum colony diameter was recorded at 25 °C, reaching 90.00 mm, suggesting that 25 °C was the optimal growth temperature among the eight temperature gradients tested. However, growth inhibition occurred when temperatures fell below 10 °C or exceeded 35 °C. Temperatures of 40 °C and above significantly impaired pathogen growth (Figure 6B). No mycelial growth was observed after 10 min in a water bath at 60 °C and 65 °C, indicating a lethal temperature of approximately 60 °C for the pathogen. The pathogen demonstrated the ability to grow in media with pH values ranging from 3 to 11 (Figure 6C), with a notable difference (*p* < 0.05). Colonies in pH 3 media reached 90.00 mm by the third day, faster than in other treatment groups. Overall, pathogen colony size decreased as pH increased, indicating that the pathogen favored acidic conditions. Light exposure had a significant impact on pathogen growth (Figure 6D), with colonies exhibiting larger diameters in light than in darkness. Various carbon and nitrogen sources also significantly influenced pathogen growth, with the largest colony size observed when carboxymethylcellulose sodium was used as the carbon source (Figure 6E), and when casein tryptone served as the nitrogen source (Figure 6F).

### 2.6. Screening of In Vitro Fungicides

The concentration for 50% of the maximal effect (EC50) refers to the concentration required to achieve 50% of the maximum inhibitory effect. The inhibitory impact of each fungicide on pathogen growth can be quantitatively assessed by establishing a toxicity regression equation for each fungicide and calculating its EC50. The results (Table 2 and Figure S2) showed that 70% thiophanate-methyl exhibited the lowest EC50 at 0.0169 μg/mL, followed by 43% tebuconazole (EC50 = 0.0219 μg/mL), 20% xinjunan acetate (EC50 = 0.0271 μg/mL), and 40% difenoconazole (EC50 = 0.0954 μg/mL). These findings indicate that all four fungicides can exert a potent inhibitory effect on pathogen growth at relatively low concentrations.

### 2.7. Validation of Field Efficacy

Field application results (Table 3) revealed that the morbidity of *I. polycarpa* treated with sprays containing 43% tebuconazole and 20% xinjunan acetate were 50.74% and 57.55%, respectively, both significantly lower than the control group (water). This suggests that these two fungicides have a strong inhibitory effect on the progression of canker lesions. The disease index provides a comprehensive measure of disease severity in plants. The results showed that the disease index for *I. polycarpa* sprayed with 43% tebuconazole was the lowest (11), while 70% thiophanate-methyl showed the highest disease index (16), both lower than the control group’s index of 17. This indicates that the application of fungicides had a beneficial effect in alleviating the disease in *I. polycarpa*. When considering the overall control efficacy of the fungicides on *I. polycarpa* canker, the average efficacy values were as follows: 43% tebuconazole (35.29%) > 40% difenoconazole (25.53%) > 20% xinjunan acetate (11.76%) > 70% thiophanate-methyl (5.82%). No phytotoxic effects on the growth of *I. polycarpa* were observed during the trial.

## 3. Discussion

As a high-quality woody oilseed tree, *I. polycarpa* has been widely planted. This study investigated two plantation forests and revealed that canker disease poses a serious threat to *I. polycarpa*. It impairs growth in the early stages and leads to death in severe stages, potentially jeopardizing the large-scale cultivation of *I. polycarpa*. The major pathogen of *I. polycarpa* canker was identified through conventional tissue isolation and Koch’s postulates. Morphological observations and multigene sequence analysis confirmed the pathogen as *Botryosphaeria dothidea*. *B. dothidea* is widespread and has been documented in several countries, including China [20], Japan [21], the United States [22], Iran [23], Spain [24], and Italy [25]. It infects a variety of woody host plants globally, such as *Cinnamomum camphora* [26], Taxus × media [27], *Persea americana* [3], *Ficus microcarpa* [28], *Carya cathayensis* [29], and *Prunus persica* [30]. Our observations also indicated that *I. polycarpa* canker primarily occurs on the tree’s stem, and as the disease progresses, the cankers spread from the stem to the branches. This suggests that *I. polycarpa* canker is a systemic and highly damaging disease. Previous studies have shown that canker disease pathogens can induce tree dieback by causing functional failure of the phloem and cambium and carbon starvation in the xylem [31]. However, whether the mechanism by which *B. dothidea* kills *I. polycarpa* is similar remains to be further explored.

Based on field surveys, we observed a consistent pattern in the occurrence of *I. polycarpa* canker. The disease is primarily concentrated in months with favorable temperatures, sufficient light, and abundant rainfall, supporting the conclusion that the pathogen grows most rapidly at 25 °C and under full light conditions. *B. dothidea* is a weakly parasitic pathogen, characterized by latent infestation [32]. It typically resides in the soil as a saprophyte or within healthy plants as an endophyte. When favorable environmental conditions arise, it initiates plant infestation [33]. *Pistacia vera* is particularly susceptible to *B. dothidea* infection during the rainy season [34], and walnut shoot blight typically occurs in the warm, wet months of July and August [35]. Suitable environmental conditions promote pathogen growth and disease occurrence. Therefore, understanding the biological characteristics and growth patterns of pathogens is crucial for effective disease control and prevention in the field, and it provides a solid foundation for scientific management strategies.

In this study, the *B. dothidea* strain SQ5 exhibited strong aggressiveness toward *I. polycarpa*. Inoculation experiments showed rapid lesion expansion, tissue necrosis, and in some cases plant wilting and death, fulfilling Koch’s postulates. Compared to reports of *B. dothidea* infecting other woody hosts, the SQ5 strain showed similarly high virulence, indicating its strong ability to colonize and damage *I. polycarpa* tissues. The aggressiveness may be attributed to its optimal growth at 25 °C, tolerance to acidic pH, broad carbon/nitrogen utilization, and a relatively high mycelial lethal temperature (60 °C), all of which enhance its adaptability and infection potential under field conditions. These traits make SQ5 a key pathogen of concern in *I. polycarpa* plantations and underscore the necessity for targeted control strategies.

In this study, we found that the *I. polycarpa* canker pathogen can utilize various carbon and nitrogen sources, indicating its capacity to thrive in diverse environmental conditions and survive under a wide range of circumstances, consistent with previous research [35,36,37]. However, we also observed some notable differences: the pathogen prefers an acidic environment but can still grow under alkaline conditions. This contrasts with the *B. dothidea* pathogen of *Osmanthus fragrans* leaf spot, which ceases growth at pH 3 [38], and the *B. dothidea* pathogen of *Photinia serrulata* leaf blight, which exhibits optimal growth at pH 6 [19]. Additionally, the mycelial lethal temperature in this study was 60 °C, higher than the mycelial lethal temperatures of 50 °C for the sweet cherry gum disease pathogen (*B. dothidea*) [39] and 52 °C for the passion fruit stem rot pathogen (*B. dothidea*) [40]. After plant infestation, during long-term parasitism and influenced by genetic, host, and environmental factors, the pathogenicity, cultural phenotype, and biological characteristics of the pathogen may exhibit significant variation between individuals [41,42,43]. This suggests that the *B. dothidea* strain in this study may have undergone mutation. Moreover, with global warming, the increasing frequency of warm weather may heighten the likelihood of *I. polycarpa* canker outbreaks.

When plants are infected with canker, they gradually exhibit a range of symptoms, including stem blight, ulcers, gum secretion, and fruit rot, as the pathogen’s growth progresses, which adversely impacts plant health [44,45]. Currently, various measures are available to control the disease, including biological, chemical, and physical methods. Chemical control is widely used in agricultural production due to its simplicity and high efficiency [46]. Numerous fungicides are available on the market, each with varying levels of effectiveness against different plant diseases and pathogens. In vitro screening eliminates the influence of environmental factors and focuses on the fungicides’ direct impact on pathogens, offering precise insights for pathogen control [47]. Li et al. [48] selected four fungicides from a pool of sixteen that showed significant inhibitory activity against the *Myrica rubra* twig blight pathogen. Similarly, Denman et al. [49] identified several fungicides, including tebuconazole, benomyl, and prochloraz, through laboratory experiments, providing valuable guidance for the field control of *Protea magnifica* canker. In this study, we screened four effective fungicides—70% thiophanate-methyl, 43% tebuconazole, 20% xinjunan acetate, and 40% difenoconazole—using a plate inhibition test. The EC50 values were 0.0169 μg/mL, 0.0219 μg/mL, 0.0271 μg/mL, and 0.0954 μg/mL, respectively. These fungicides also demonstrated strong inhibition of canker disease in other plants. For instance, Jiang et al. [50] found that 70% thiophanate-methyl had a strong inhibitory effect (EC50 = 9.3 μg/mL) on *B. dothidea* in their study on Chinese hickory canker. Tebuconazole inhibited the growth of *B. dothidea* (California pistachio blight) at low concentrations, with EC50 values ranging from 0.019 to 0.159 μg/mL [51]. Difenoconazole exhibited potent inhibitory activity against peach twig blight pathogens (*Phomopsis amygdali*, *Botryosphaeria obtusa*, and *Leucostoma persoonii*) with EC50 values of 0.289 μg/mL, 0.026 μg/mL, and 0.858 μg/mL, respectively [52]. The results from this study provide valuable insights for controlling *I. polycarpa* canker in the field.

During the in vitro fungicide screening, the fungicides showed strong inhibition through direct contact with the pathogens. However, the efficacy of these fungicides may be influenced by natural environmental conditions and the specific characteristics of the disease. Therefore, additional trials are required to assess and confirm their effectiveness against *I. polycarpa* canker under field conditions.

To assess the effectiveness of the fungicides under field conditions, we conducted a field validation trial. The results showed that, among the four fungicides tested, 43% tebuconazole and 20% Xinjunan acetate effectively reduced the incidence of *I. polycarpa* canker. Tebuconazole has been widely used to control plant cankers, reducing the occurrence of canker diseases caused by *Botryosphaeriales* fungi in apple and pear by 20% [53] and the incidence of Australian grapevine canker in the field by 41–65% [54]. In contrast, 40% difenoconazole had no significant effect on the incidence of *I. polycarpa* canker in our study, although previous research reported a 40% reduction in the incidence of apple necrotic rot [55]. In terms of tree health, the average efficacy of 43% tebuconazole and 40% difenoconazole against *I. polycarpa* canker in our study was 35.29% and 25.53%, respectively, which was lower than the control efficacy reported for other plant canker diseases. However, the field performance of 20% xinjunan acetate and 70% thiophanate-methyl was low, with control effects significantly lower than those observed in the laboratory tests. Olmo et al. [56] found that thiophanate-methyl significantly inhibited the apricot canker pathogen (*Botryosphaeriaceae* spp.) in vitro, with EC50 values ranging from 0.65 to 0.77 mg/L, and confirmed its effectiveness in reducing the length of apricot canker lesions and the pathogen isolation rate in field trials. The efficacy of fungicides is closely related to environmental factors such as temperature, moisture, and wind, which can reduce the concentration of active ingredients and affect treatment effectiveness [57]. Additionally, the timing and method of application can also influence efficacy. For example, spraying fungicides on peach trees in autumn can reduce canker incidence by 45–63%, while spring applications only reduce incidence by 10–28% [58]. Scraping lesions before applying fungicide has been shown to improve the control of *Litchi chinensis* canker [59]. Furthermore, Miller et al. [60] found that mixing fungicides can significantly improve efficacy. For instance, combining thiophanate-methyl with VitiSeal reduced peach canker pathogen viability more effectively than single applications. Our field trial, conducted in Zhengzhou City from July to August, occurred under climatic conditions characterized by high average daily temperatures and frequent rainfall. These factors may have compromised the effectiveness of the sprayed fungicides. Additionally, *I. polycarpa* canker originates from beneath the bark, where the pathogen resides, potentially reducing the fungicide’s ability to penetrate and effectively control the disease. To date, no research has specifically addressed field control methods for *I. polycarpa* canker. Based on these findings, future research should focus on optimizing fungicide application methods and formulations to improve the field control of *I. polycarpa* canker.

## 4. Materials and Methods

### 4.1. Investigation of Field Diseases 

Numerous *I. polycarpa* trees were planted at two locations: the Forest Seedling Breeding Engineering Technology Center of Henan Agricultural University (FSB) (113°38′39″, 34°48′2″) and the Science and Education Park of Henan Agricultural University (SEP) (113°36′, 34°50′24″) (Figure 6). The ages of the trees range from 3 to 15 years. Over time, as the duration of planting increased, symptoms of canker disease were observed at both locations, worsening each year. Therefore, between 2020 and 2021, we conducted field surveys to observe and record the timing and symptoms of *I. polycarpa* canker to better understand its incidence pattern.

### 4.2. Pathogen Collection and Isolation

During the *I. polycarpa* canker outbreak, 20 plants exhibiting typical canker symptoms were selected from each of the two sites for symptomatic sample collection. These samples were transported to the laboratory at the College of Forestry, Henan Agricultural University, for further analysis. For culture isolation, small sections (0.5 cm × 0.5 cm) of symptomatic stem tissue were surface-disinfected in 0.10% mercuric chloride for 30 s, transferred to 75% ethanol for another 30 s, rinsed four times in sterile water, dried on sterile absorbent paper, and then placed on potato dextrose agar (PDA) (Beijing Aoboxing Universeen Bio-tech Co., Ltd., Beijing, China) plates (90 mm in diameter) supplemented with 50 mg/L ampicillin (Sigma-Aldrich, St. Louis, MO, USA) to inhibit bacterial growth [2]. The plates were incubated at 25 ± 1 °C. The pathogen was purified by subculturing the colony edges after 4–6 days of growth, ensuring that the colonies had expanded but had not yet confluenced. The purified pathogen was then preserved using slant culture medium [61].

### 4.3. Pathogenicity Testing

To identify the primary pathogens causing *I. polycarpa* canker, the pathogenicity of the isolated strains was assessed following Koch’s postulates. Pathogens from the slant medium were inoculated onto PDA plates and incubated at 25 ± 1 °C in the dark for 3 days to activate the culture. Subsequently, 5 mm mycelial plugs were extracted from the actively growing margin of the colony using a sterile hole punch. A small wound was made by removing a piece of bark (~5 mm in diameter) from the stem using a sterile scalpel. Healthy 3-year-old *I. polycarpa* seedlings were selected. An activated fungal plug was inserted into each wound. Each isolate was inoculated onto three seedlings, which were then wrapped tightly with sterile cotton saturated in sterile water and secured with parafilm. A 5 mm agar plug without the isolate served as the control. Symptoms were observed and recorded 21 days post-inoculation.

### 4.4. Morphological and Cultural Characterization

The pathogen was inoculated onto PDA plates and incubated at 25 ± 1 °C for 5 days, with colony morphology observed and recorded daily. The activated fungal isolate was also cultured on Murashige and Skoog (MS) culture medium, a plant tissue culture medium commonly used to promote sporulation in certain fungi [62]. Fresh cedar pine needles were rinsed in sterile water, evenly spread over the medium, and incubated in the dark at 25 ± 1 °C to promote sporophore and conidia formation [63]. The lengths and widths of 30 conidia were measured, and the shapes of both conidiophore and conidia were observed using an automated upright fluorescence microscope (Axio Imager M2, Carl Zeiss AG, Germany) with ZEN 3.8 software (Carl Zeiss AG, Oberkochen, Germany).

### 4.5. Molecular Identification

Approximately 100 mg of mycelium, activated at 25 ± 1 °C for 3 days, was collected and ground into a fine powder in a mortar using liquid nitrogen. Genomic DNA was then extracted using the Rapid Fungi Genomic DNA Isolation Kit (Sangon Biotech Co., Ltd., Shanghai, China), following the manufacturer’s instructions. The ITS, β-*tubulin*, and translation elongation factor 1-α (*tef1*-α) genes were amplified and sequenced following established protocols [64,65,66]. PCR primers for the respective genes included: ITS1 (5′-TCCGTAGGTGAACCTGCGG-3′), ITS4 (5′-CCTCCGCTTATTGATATGC-3′) [67], Bt2a (5′-GGTAACCAAATCGGTGCTGCTTTC-3′), Bt2b (5′-ACCCTCAGTGTAGTGACCCTTGGC-3′) [68], EF1-728F (5′-CATCGAGAAGTTCGAGAAGG-3′), and EF1-986R (5′-ACTTGAAGGAACCCTTACC-3′) [69]. Although primer specificity assays were not conducted in this study, all primers used for the amplification of ITS, β-*tubulin*, and *tef1*-α regions were selected based on previously published and widely validated protocols. These primers have been extensively applied in the molecular identification of fungal pathogens, including *B. dothidea*.

PCR was conducted in a PCR apparatus (T100, Bio-Rad Laboratories, Hercules, CA, USA) with a 25 μL reaction mixture comprising 1 μL each of upstream and downstream primers, 2 μL fungal DNA template, 0.25 μL Taq DNA polymerase, 1 μL dNTPs, 2.5 μL 10X buffer, and 18.25 μL ddH_2_O. The PCR amplification conditions were as follows: pre-denaturation at 94 °C for 10 min, denaturation at 94 °C for 30 s, annealing at 55–56 °C for 30 s, extension at 72 °C for 30 s, and a final extension at 72 °C for 10 min, repeated for 35 cycles. Agarose gel electrophoresis (1%) in 1 × TAE buffer was used to verify successful PCR amplification. Subsequently, DNA sequencing was performed by Beijing Liuhe Huada Gene Technology Co., Ltd., Beijing, China. Sequences obtained in this study were deposited in GenBank, and the accession numbers for the isolates are listed in Table 1.

### 4.6. Phylogenetic Analysis

ITS, β-*tubulin*, and *tef1*-α sequences of strains with high similarity to those in this study (including the ex-type strain) were selected based on correlation studies [17] and BLAST + 2.16.0 (Basic Local Alignment Search Tool) searches in the NCBI nucleotide database. DNA sequence datasets for *ITS*, β-*tubulin*, and *tef1*-α were aligned using MEGA 11 (Mega Limited, Auckland, New Zealand) [70], with manual adjustments as needed. Phylogenetic analyses of the combined ITS + *tef1*-α + β-*tubulin* dataset were conducted using PAUP v.4.0 (Sinauer Associates, Sunderland, MA, USA) [28]. Both Maximum Parsimony (MP) and Maximum Likelihood (ML) methods were employed. For the MP analysis, the heuristic search function and tree bisection and reconstruction (TBR) were used as the branch-swapping algorithm, with the branch-swapping option set to ‘best tree’ only. Gaps were treated as ‘missing’, and characters were unordered and equally weighted [25], with Maxtrees limited to 100. Tree length (TL), consistency index (CI), retention index (RI), and rescaled consistency index (RC) were calculated. Clade support in both analyses was evaluated through 1000 bootstrap replicates. Additionally, *Tiarosporella graminis* (strain CBS 118718; GenBank ITS = KC769962, BT = KF531808, TEF = KF531807) was chosen as an outgroup [17].

### 4.7. Research on Biological Characteristics

The pathogen was activated on PDA plates at 25 ± 1 °C for 3 days. Mycelial plugs (5 mm diameter) were excised from the actively growing colony margin using a sterile hole punch and subjected to the treatments described below. Each treatment included three replicates. Colony diameters were measured after 5 days of incubation using the cross-measurement method [25].

Effect of Temperature on Pathogen Growth: Mycelial plugs were cultured onto PDA plates and incubated in a constant temperature chamber at 5 °C, 10 °C, 15 °C, 20 °C, 25 °C, 30 °C, 35 °C, and 40 °C in the dark for 5 days.

Determination of the Lethal Temperature of the Pathogen: Each mycelial plug was placed in a 10 mL sterile centrifuge tube with 5.00 mL of sterile water. Tubes were immersed in a water bath at 40 °C, 45 °C, 50 °C, 55 °C, 60 °C, and 65 °C for 10 min [71]. After cooling to room temperature, each mycelial plug was inoculated onto a PDA plate and incubated at 25 ± 1 °C for 5 days.

Effect of pH on Pathogen Growth: The pH of the PDA medium was adjusted to 3, 4, 5, 6, 7, 8, 9, 10, and 11 using 1.00 mol/L HCl and 1.00 mol/L NaOH [72]. mycelial plugs were then inoculated onto PDA plates of each pH level and incubated at 25 ± 1 °C for 5 days.

Effect of Light on Pathogen Growth: Mycelial plugs were inoculated onto PDA plates and incubated at 25 ± 1 °C under four lighting conditions: complete light, complete darkness, and alternating 12-h cycles of light and darkness for 5 days.

Effect of Carbon and Nitrogen Sources on Pathogen Growth: Czapek-Dox medium was used as the base. Sucrose in the medium was replaced with glucose, fructose, galactose, mannitol, sorbitol, lactose, carboxymethylcellulose sodium, or xylose to create different carbon source media. Sodium nitrate was replaced with ammonium chloride, potassium nitrate, urea, casein tryptone, ammonium acetate, ammonium sulfate, ammonium oxalate, or ammonium dihydrogen phosphate to create different nitrogen source media [72]. Each mycelial plug was inoculated onto these carbon and nitrogen source media and incubated at 25 ± 1 °C for 5 days.

### 4.8. In Vitro Fungicide Screening

To evaluate the effectiveness of fungicides in inhibiting pathogen growth, we tested 17 commercially available fungicides commonly used for controlling forest cankers (Table S1). A concentration gradient was established based on the recommended concentration for each fungicide, and each fungicide was mixed with PDA medium in a 1:9 ratio to create a fungicide-containing medium [28]. Activated mycelial plugs were then inoculated into each fungicide-containing medium, while PDA medium without fungicide served as the control. Three replicates were prepared for each treatment, and all plates were incubated in the dark at 25 ± 1 °C for 5 days. Colony diameters on each plate were measured using the crossover method, and the antifungal rate was calculated according to Equation (1).

(1) $$\begin{array}{c} IR=\frac{(\mathrm{DCC}-\mathrm{DC})-(\mathrm{DTC}\;-\;\mathrm{DC})}{\mathrm{DCC}-\mathrm{DC}}\times 100 \end{array}$$

IR, Inhibition rate; DCC, Diameter of control colony; DC, Diameter of mycelial plug; DTC, Diameter of treated colonies.

### 4.9. Methods for Field Efficacy Verification

To assess the efficacy of fungicides screened in vitros, field trials were conducted. Diseased *I. polycarpa* trees of the same seed source, similar age, and growth were selected from the two trial sites, and the disease status of these sample trees was observed and recorded. Fungicides were chosen based on their low EC50 values obtained from in vitro toxicity measurements (Table 1), with concentrations set to achieve > 90% pathogen inhibition (Table S2). Five treatment groups were established according to the specifications in Table 4, with five replicates per treatment. Each group was kept separate to maintain experimental integrity.

The first application of the spray was carried out on 6 July 2022, using a hand-pressurized sprayer, followed by subsequent applications on 13, 20 and 27 July, with 1 L of each fungicide solution applied each time. The disease status of the sample trees was recorded on 3 August. Visual counts were conducted for spots on all sample trees, and the breast diameter of each tree was measured using a breast diameter caliper, along with the size of each spot. Disease ratings were calculated for each tree based on Table 5 [53,54,73]. Morbidity, disease index, and average efficacy were computed according to Equations (2), (3), and (4), respectively. Field efficacy tests were conducted following the EPPO guideline PP 1/135(4) for fungicide evaluation under practical conditions [74].

(2) $$M=\frac{\mathrm{NSBT}-\mathrm{NSAT}}{\mathrm{NSAT}}\times \;100$$

M: Morbidity; NSBT: Number of spots before treatment; NSAT: Number of spots after treatment.

(3) $$\mathrm{DI}=\frac{\sum \left(\mathrm{NPPL}\;\times \;\mathrm{LRV}\right)}{\mathrm{TNPS}\;\times \;\mathrm{HRV}}\times \;100$$

DI: Disease index; NPPL: Number of plants per level; LRV: This level’s representative value; TNPS: Total number of plants surveyed; HRV: Highest representative value.

(4) $$\mathrm{AE}=\frac{\mathrm{CGDI}-\mathrm{TGDI}}{\mathrm{CGDI}}\times \;100$$

AE: Average efficacy; CGDI: Control group disease index; TGDI: Treatment group disease index.

**Table 5:** Disease severity grading scale for field evaluation of *I. polycarpa* canker.

Disease Rate	Representative Number	Grading Standard
I	0	No lesions
II	1	1 to 10 spots on the main stem
III	2	The main stem has more than 10 spots and the width of the largest spot is less than 1/4 of the circumference of the tree.
IV	3	The main stem has more than 10 spots and the width of the largest spot is up to 1/4 but not more than 1/2 of the circumference of the tree.
V	4	The stem or branches are covered with disease scars, the maximum width of the spot is more than 1/2 the circumference of the tree, the branches and stems are crippled and the tree is weakened or even dead.

### 4.10. Statistical Analysis

Data were processed and statistical analyses were performed using Excel 2021 (Microsoft Corp., Redmond, WA, USA). Normality and homogeneity of variances were assessed, and data were log2-transformed when necessary prior to analysis. Analysis of variance (ANOVA) was conducted using the ‘aov’ function in R (R Foundation for Statistical Computing, Vienna, Austria), followed by Tukey’s honestly significant difference (HSD) test for post hoc comparisons. Visualizations were generated using the ‘ggplot2’ package in R. The spatial distribution of sampling sites was mapped using ArcGIS 10.7 (ESRI Inc., Redlands, CA, USA).

## 5. Conclusions

This study provides the first systematic report of canker disease in *I. polycarpa*, identifying *Botryosphaeria dothidea* as the causal agent based on morphological and molecular evidence and confirming its pathogenicity via Koch’s postulates. The pathogen exhibits strong environmental adaptability and poses a significant threat to *I. polycarpa* plantations. In vitro and field evaluations revealed that 43% tebuconazole offers the most effective control among the tested fungicides. These findings lay a foundation for future research on disease epidemiology and management and highlight the need for integrated strategies to mitigate the impact of this emerging disease in expanding *I. polycarpa* cultivation.

## Figures and Tables

**Table 1:** Sequences for phylogenetic analysis.

Species	Isolate ID	*ITS*	*tef1-α*	β-*Tubulin*
**SQ5**	**STZ-1 = STZ3 = STZ2**	**OR523573**	**OR542956**	**OR542955**
*Neofusicoccum parvum*	CBS 123650	KX464182	KX464708	KX464994
*Neofusicoccum parvum*	CBS 145622	MN611179	MN623346	MN623343
*Neofusicoccum parvum*	CBS 145623	MN611180	MN623347	MN623344
*Botryosphaeria parva* *	CMW9077	AY236939	AY236884	AY236913
*Botryosphaeria parva* *	CMW9081	AY236943	AY236888	AY236917
*Botryosphaeria ribis* *	CMW7772	AY236935	AY236877	AY236906
*Botryosphaeria ribis* *	CMW7773	AY236936	AY236878	AY236907
*Botryosphaeria lutea*	KJ 93.52 = CMW992 = CMW992	AF027745	AY236894	AY236923
*Botryosphaeria lutea*	CMW9076	AY236946	AY236893	AY236922
*Botryosphaeria dothidea* *	CMW8000	AY236949	AY236898	AY236927
*Botryosphaeria dothidea* *	CMW7999	AY236948	AY236897	AY236926
*Botryosphaeria dothidea*	JZG1	KU360149	KU565872	KU565871
*Botryosphaeria dothidea*	JZB310203	MN945382	MT269886	MT371073
*Botryosphaeria dothidea*	JZB310200	MN945379	MT269883	MT371070
*Tiarosporella graminis*	CBS 118718	KC769962	KF531807	KF531808

Sequences in this study are marked in bold. The ex-type strain is marked with *.

**Table 2:** Toxicity test results of fungicides to pathogen.

Number	Medicament	Toxicity Regression Equation	R^2^	EC50 (μg/mL)
1	40% Thiabendazole	y = 0.9782x + 4.3588	0.9996	4.5237
2	Azoxystrobin	y = 0.358x + 4.2849	0.7922	99.4288
3	30% Metalaxyl-M-metidaxyl	y = 2.1806x + 2.7381	0.9348	10.8964
4	43% Tebuconazole	y = 1.4269x + 7.369	0.9689	0.0219
5	20% Thiediazole copper	y = 1.9096x + 3.5989	0.9829	5.4164
6	Lime sulfur	y = 0.2771x + 4.3538	0.5997	214.7880
7	20% Xinjunan acetate	y = 0.0316x + 6.0327	0.9376	0.0271
8	36%Kasugamycin-oxine-copper	y = 1.5122x + 4.0343	0.8071	4.3512
9	40% Difenoconazole	y = 0.8937x + 5.8912	0.9658	0.0954
10	6% kasugamycin	y = 1.8315x + 3.9717	0.9861	3.6433
11	70% Thiophanate-methyl	y = 0.8978x + 6.5902	0.9918	0.0169
12	47% Kasugamycin-copper oxychloride	y = 1.58x + 4.2467	0.9383	2.9976
13	27% Bromothalonil	y = 1.1286x + 4.0043	0.8658	7.6243
14	33.5% Oxine-copper	y = 0.3398x + 4.3141	0.9464	104.3615
15	25% Oligosaccharins-ethylicin	y = 0.9268x + 4.8564	0.9802	1.4286
16	30% Zincthiazole	y = 0.4042x + 4.534	0.9429	14.2200
17	80% Bordeaux mixture	y = 1.1139x + 4.9288	0.9561	1.1585

**Table 3:** Control effect of *I. polycarpa* canker disease in the field.

Medicament	Concentration (μg/mL)	Morbidity (%)	Disease Index	Average Efficacy (%)	Security
43% Tebuconazole	0.5	50.74 ± 5.23 c	11	35.29	Lower toxicity
20% Xinjunan acetate	2	57.55 ± 6.05 bc	15	11.76	Lower toxicity
40% Difenoconazole	4.5	70.00 ± 5.97 ab	13	23.53	Lower toxicity
70% Thiophanate-methyl	3.3	73.06 ± 1.06 a	16	5.82	Lower toxicity
Water (CK)	\	79.20 ± 1.49 a	17	\	Innocuity

Note: Different letters show significant differences among groups (mean ± SE, *p* < 0.05, n = 5).

**Table 4:** Test fungicide’s information and concentration.

Fungicide	Manufacturer	Concentration (mg/L)
43% Tebuconazole	Bayer CropScience China Co., Ltd., Beijing, China	0.50
20% Xinjunan acetate	Hebei Shenhua Pharmaceutical Co., Ltd., Shijiazhuang, China	2.00
40% Difenoconazole	Shanghai Hulian Biopharmaceutical (Xiayi) Co., Ltd., Shanghai, China	4.50
70% Thiophanate-methyl	Xi’an Dingsheng Biochemical Co., Ltd., Xi’an, China	0.63
Water	\	\

## Data Availability

All raw sequencing reads generated were deposited in the NCBI Sequence Read Archive (Nucleotide) under submission accession number OR523573, OR542956 and OR542955.

## Supplementary Materials

The following supporting information can be downloaded at: https://www.mdpi.com/article/10.3390/plants14091393/s1. Figure S1: Average monthly temperature and precipitation in Zhengzhou from 2021 to 2022; Figure S2: In vitro control using 17 different fungicides. The datasets generated and analyzed during the current study are available in the Figshare repository (available at: https://doi.org/10.6084/m9.figshare.28852358), Table S1: 17 Fungicide information. Table S2: Fungicide’s antibactenial rate.

## References

[1] López-López Á., Allende-Molar R., Correia K.C., Tovar-Pedraza J.M., Márquez-Zequera I., García-Estrada R.S. (2021). First report of stem canker of Tomato caused by *Fusarium striatum* in Mexico. Plant Dis..

[2] Kovač M., Diminić D., Orlović S., Zlatković M. (2021). *Botryosphaeria dothidea* and Neofusicoccum yunnanense causing canker and die-back of Sequoiadendron giganteum in Croatia. Forests.

[3] Hernández D., García-Pérez O., Perera S., González-Carracedo M.A., Rodríguez-Pérez A., Siverio F. (2023). Fungal pathogens associated with aerial symptoms of avocado (*Persea americana* Mill.) in Tenerife (Canary Islands, Spain) focused on species of the family Botryosphaeriaceae. Microorganisms.

[4] Kenfaoui J., Lahlali R., Mennani M., Radouane N., Goura K., El Hamss H., El Ghadraoui L., Fontaine F., Tahiri A., Barka E.A. (2022). Botryosphaeria dieback (Lasiodiplodia viticola): An imminent emerging threat to the Moroccan vineyards. Plants.

[5] Houston D.R., Valentine H.T. (1988). Beech bark disease: The temporal pattern of cankering in aftermath forests of Maine. Can. J. For. Res..

[6] Ruess R.W., Winton L.M., Adams G.C. (2021). Correction: Widespread mortality of trembling aspen (*Populus tremuloides*) throughout interior Alaskan boreal forests resulting from a novel canker disease. PLoS ONE.

[7] Chen W.Y., Jiang N., Fan X.L., Guo H.L., Tian C.M. (2020). Preliminary identification of pathogenic fungal groups from *Pinus armandii* canker in Gansu Province. J. Northwest For. Univ..

[8] Lv Q., Zhang Y.F., Lin R.Z., Wang H.M. (2022). Occurrence status of main forestry invasive species in China and their research trends. Plant Prot..

[9] Wang H., Rana S., Li Z., Xu G., Wang Y., Cai Q., Li S., Sun J., Liu Z. (2022). Morphological and anatomical changes during floral bud development of the trioecious *Idesia polycarpa* Maxim. Braz. J. Bot..

[10] Tian X.X., Fang X.Z., Du M.H. (2020). Nutritional qualities and antioxidant properties of *Idesia polycarpa* Maxim. pulp oil and seed oil. J. Chin. Cereals Oils Assoc..

[11] Xiang X., Wen L., Wang Z., Yang G., Mao J., An X., Kan J. (2023). A comprehensive study on physicochemical properties, bioactive compounds, and emulsified lipid digestion characteristics of *Idesia polycarpa* var. vestita Diels fruits oil. Food Chem..

[12] Zhou D., Zhou X., Shi Q., Pan J., Zhan H., Ge F. (2022). High-pressure supercritical carbon dioxide extraction of *Idesia polycarpa* oil: Evaluation the influence of process parameters on the extraction yield and oil quality. Ind. Crops Prod..

[13] Ai Q., Ai J., Wang L. (2020). Oil grape (*Idesia polycarpa*) is included in the general food management by the state. China For. Ind..

[14] Liu Y. (2022). Analysis on the development and market prospect of oil grape (*Idesia polycarpa*) industry. China For. Ind..

[15] Zhou J., Luo J.X., Yu Y.H., Liu F.R., Huang S.H., He H.G. (2023). Major disease types of *Idesia polycarpa* in Sichuan Province and its prevention and control suggestions. J. China West Norm. Univ..

[16] Zhu Z.Y. (2010). Propagation Technology and Development and Utilization of Idesia polycarpa.

[17] Zheng X.R., Zhang M.J., Shang X.L., Fang S.Z., Chen F.M. (2020). Stem canker on Cyclocarya paliurus is caused by *Botryosphaeria dothidea*. Plant Dis..

[18] He Y., Qi Z., Li S., Zhu H., Hong N., Wang G., Wang L. (2022). Molecular characterization of a new fusarivirus infecting *Botryosphaeria dothidea*, the causal agent of pear ring rot disease. Arch. Virol..

[19] Zhang Q., Zhang Y., Shi H., Huo Y. (2023). *Botryosphaeria dothidea* causing leaf blight of *Photinia serrulata* in China. Crop Prot..

[20] Dong X.L., Cheng Z.Z., Leng W.F., Li B.H., Xu X.M., Lian S., Wang C.X. (2021). Progression of symptoms caused by *Botryosphaeria dothidea* on apple branches. Phytopathology.

[21] Ono Y., Chatasiri S., Tanaka E. (2020). Ochropsora staphyleae, a new rust pathogen of Japanese bladdernut, found in central Japan. Mycoscience.

[22] Romero-Cuadrado L., López-Herrera C.J., Aguado A., Capote N. (2023). Duplex real-time PCR assays for the simultaneous detection and quantification of Botryosphaeriaceae species Causing canker diseases in woody crops. Plants.

[23] Sohrabi M., Mohammadi H., León M., Armengol J., Banihashemi Z. (2020). Fungal pathogens associated with branch and trunk cankers of nut crops in Iran. Eur. J. Plant Pathol..

[24] Gramaje D., Agustí-Brisach C., Pérez-Sierra A., Moralejo E., Olmo D., Mostert L., Damm U., Armengol J. (2012). Fungal trunk pathogens associated with wood decay of almond trees on Mallorca (Spain). Persoonia Mol. Phylogeny Evol. Fungi.

[25] Gusella G., Giambra S., Conigliaro G., Burruano S., Polizzi G. (2020). Botryosphaeriaceae species causing canker and dieback of English walnut (Juglans regia) in Italy. For. Pathol..

[26] Feng X., Lu L., Jia Y., Yan X., Zhongzhu P., Wang G., Qiu C., Wu H. (2023). First report of trunk canker caused by *Botryosphaeria dothidea* on *Cinnamomum camphora* in China. Plant Dis..

[27] Wang Y., Chen J., Lan X.X., Ren M., Zhang B. (2023). First report of shoot and leaf blight caused by *Botryosphaeria dothidea* on *Taxus × media* in Sichuan Province, China. Plant Dis..

[28] Fiorenza A., Aiello D., Costanzo M.B., Gusella G., Polizzi G. (2022). A new disease for europe of *Ficus microcarpa* caused by Botryosphaeriaceae species. Plants.

[29] Zhuang C.J., Wang Q.W., Wu Q.Q., Qiu Z.L., Xu B.C., Zhang C.Q. (2021). Diversity of Botryosphaeriaceae species associated with Chinese hickory tree (*Carya cathayensis*) trunk cankers. Plant Dis..

[30] Tian Y., Zhao Y., Sun T., Wang L., Liu J., Ma X., Hu B. (2018). Identification and characterization of *Phomopsis amygdali* and *Botryosphaeria dothidea* associated with peach shoot blight in Yangshan, China. Plant Dis..

[31] Xing J., Li P., Zhang Y., Li J., Liu Y., Lachenbruch B., Su X., Zhao J. (2020). Fungal pathogens of canker disease trigger canopy dieback in poplar saplings by inducing functional failure of the phloem and cambium and carbon starvation in the xylem. Physiol. Mol. Plant Pathol..

[32] Leal C., Trotel-Aziz P., Gramaje D., Armengol J., Fontaine F. (2023). Exploring some factors conditioning the expression of Botryosphaeria dieback in grapevine for an integrated management of the disease. Phytopathology.

[33] Slippers B., Wingfield M.J. (2007). Botryosphaeriaceae as endophytes and latent pathogens of woody plants: Diversity, ecology and impact. Fungal Biol. Rev..

[34] Ntahimpera N., Driever G.F., Felts D., Morgan D.P., Michailides T.J. (2002). Dynamics and pattern of latent infection caused by Botryosphaeria dothidea on Pistachio Buds. Plant Dis..

[35] López-Moral A., Lovera M., Raya M.D., Cortés-Cosano N., Arquero O., Trapero A., Agustí-Brisach C. (2020). Etiology of branch dieback and shoot blight of English walnut caused by Botryosphaeriaceae and Diaporthe species in southern Spain. Plant Dis..

[36] Rezgui A., Ghnaya-Chakroun A.B., Vallance J., Bruez E., Hajlaoui M.R., Sadfi-Zouaoui N., Rey P. (2016). Endophytic bacteria with antagonistic traits inhabit the wood tissues of grapevines from Tunisian vineyards. Biol. Control.

[37] Chen W., Xie F., Tian C., Li F., Chou G. (2023). Identification and characterization of Neocosmospora silvicola causing canker disease on *Pinus armandii* in China. Plant Dis..

[38] Li Y.L., Zhou Z., Yuan C.Y., Duan P.L. (2014). Biological characteristics of the leaf spot lesions of Osmanthus fragrans and its resticide sensitivity. North. Hortic..

[39] Zhang Q., Li Y., Yang P., Zhang J.H., Ma Z.H., Zhang L.Z. (2020). Biological characteristics of Botryosphaeria dothoidea from sweet cherry. Sci. Technol. Food Ind..

[40] Huang Y.H., Ning P., Huang Y.G., Ou S.S., Zhang Y.X., Tan C.N., Meng C. (2022). Identification and biological characterization of stem rot pathogens from passion fruit. Southwest China J. Agric. Sci..

[41] Sun J.E., Meng C.-R., Phillips A.J.L., Wang Y. (2022). Two new Botryosphaeria (Botryosphaeriales, Botryosphaeriaceae) species in China. MycoKeys.

[42] Li T., Li N., Lei Z., Zhang C. (2023). Sensitivity and resistance risk of Botryosphaeria dothidea causing Chinese hickory trunk canker to fludioxonil. Pestic. Biochem. Physiol..

[43] Manetti G., Brunetti A., Lumia V., Sciarroni L., Marangi P., Cristella N., Faggioli F., Reverberi M., Scortichini M., Pilotti M. (2023). Identification and characterization of Neofusicoccum stellenboschiana in branch and twig dieback-affected *Olea europaea* trees in Italy and comparative pathogenicity with *N. mediterraneum*. J. Fungi.

[44] Geng X., Liu Y., Li J., Li Z., Shu J., Wu G. (2022). Identification and characterization of Nectria pseudotrichia associated with camellia canker disease in China. Forests.

[45] Dobry E., Rutter M.A., Campbell M.A. (2023). The fungal pathogen Gnomoniopsis castaneae induces damaging cankers in multiple domestic Fagaceae Species. Phytopathology.

[46] Feng H., Wang C., Yang H., Tang L., Han P.-L., Liang J., Huang L. (2023). Apple valsa canker: Insights into pathogenesis and disease control. Phytopathol. Res..

[47] Tudela M.A.A., Lutz M.C., Giménez G.N., Del Brío D., Di Masi S.N., Pose G.N., Molina J.P.E. (2023). Efficacy of fungicides against brown spot of pear in Argentina. Crop Prot..

[48] Li W., Hu M., Xue Y., Li Z., Zhang Y., Zheng D., Lu G., Wang J., Zhou J. (2020). Five fungal pathogens are responsible for bayberry twig blight and fungicides were screened for disease control. Microorganisms.

[49] Denman S., Crous P.W., Sadie A., Wingfield M.J. (2004). Evaluation of fungicides for the control of Botryosphaeria protearum on *Protea magnifica* in the Western Cape Province of South Africa. Australas. Plant Pathol..

[50] Jiang H., Rao Y., Li M., Wang Y. (2020). Antifungal activity of rapamycin on Botryosphaeria dothidea and its effect against Chinese hickory canker. Pest Manag. Sci..

[51] Ma Z., Morgan D.P., Felts D., Michailides T.J. (2002). Sensitivity of Botryosphaeria dothidea from California pistachio to tebuconazole. Crop Prot..

[52] Froelich M.H., Schnabel G. (2019). Investigation of fungi causing twig blight diseases on peach trees in South Carolina. Plant Dis..

[53] Sosa M.C., Lutz M.C., Lódolo X.V., Basso C.N. (2022). In vitro and in vivo activity of chemical fungicides and a biofungicide for the control of wood diseases caused by Botryosphaeriales fungi in apple and pear. Int. J. Pest Manag..

[54] Pitt W.M., Sosnowski M.R., Huang R., Qiu Y., Steel C.C., Savocchia S. (2012). Evaluation of fungicides for the management of Botryosphaeria canker of grapevines. Plant Dis..

[55] Song Y., Li L., Li C., Lu Z., Men X., Chen F. (2018). Evaluating the sensitivity and efficacy of fungicides with different modes of action against Botryosphaeria dothidea. Plant Dis..

[56] Olmo D., Gramaje D., Armengol J. (2017). Evaluation of fungicides to protect pruning wounds from Botryosphaeriaceae species infections on almond trees. Phytopathol. Mediterr..

[57] Hou Y., Guo Y., Wang L., He S., Zheng W., Liu S., Xu J. (2023). Impact of phenamacril on the growth and development of Fusarium pseudograminearum and control of crown rot of wheat. Plant Dis..

[58] Lalancette N., Robison D.M. (2002). Effect of fungicides, application timing, and canker removal on incidence and severity of constriction canker of peach. Plant Dis..

[59] Liao M.J., Zhang Z.H., Xi P.G., Wu Y.Z., Zhang H.L., Jiang Z.D. (2021). Incidence and control of litchi stem rot in Zengcheng. China Trop. Agric..

[60] Miller S.T., Sterle D., Minas I.S., Stewart J.E. (2021). Exploring fungicides and sealants for management of Cytospora plurivora infections in western Colorado peach production systems. Crop Prot..

[61] Zhang C.Q., Xu B.C. (2011). First report of canker on Chinese hickory (*Carya cathayensis*) caused by Botryosphaeria dothidea in China. Plant Dis..

[62] Murashige T., Skoog F. (1962). A revised medium for rapid growth and bio assays with tobacco tissue cultures. Physiol. Plant..

[63] Liu J., Zhang L.Y., Wang H.Y., Liu N., Lian S., Xu X.M., Li B.H. (2022). The effect of temperature and moisture on colonization of apple fruit and branches by Botryosphaeria dothidea. Phytopathology.

[64] White T.J., Bruns T., Lee S., Taylor J. (1990). Amplification and direct sequencing of fungal ribosomal RNA Genes for phylogenetics. PCR Protoc..

[65] Glass N.L., Donaldson G.C. (1995). Development of primer sets designed for use with the PCR to amplify conserved genes from filamentous ascomycetes. Appl. Environ. Microbiol..

[66] Alves A., Crous P., Correia A., Phillips A. (2008). Morphological and molecular data reveal cryptic speciation in Lasiodiplodia theobromae. Fungal Divers..

[67] Yu L., Wang L.F., Zhao J.R., Xu S.G., Gao D., Zheng J.F. (2012). First report of *Botryosphaeria dothidea* causing canker and dieback disease of Helwingia chinensis in China. Plant Dis..

[68] Al-Naemi F.A., Nishad R., Ahmed T.A., Radwan O. (2014). First report of *Thielaviopsis punctulata* causing black scorch disease on date palm in qatar. Plant Dis..

[69] Tang W., Ding Z., Zhou Z.Q., Wang Y.Z., Guo L.Y. (2012). Phylogenetic and pathogenic analyses show that the causal agent of apple ring rot in China is *Botryosphaeria dothidea*. Plant Dis..

[70] Tamura K., Peterson D., Peterson N., Stecher G., Nei M., Kumar S. (2011). MEGA5: Molecular evolutionary genetics analysis using maximum likelihood, evolutionary distance, and maximum parsimony methods. Mol. Biol. Evol..

[71] Shi J., Sang W.J., Lu Y.H., Zhang D.P., Kong F., Shou A.F., Yang M.M. (2023). Identification of tobacco anthracnose pathogen and its biological characteristics in Guangxi. Chin. Tob. Sci..

[72] Cui L., Yang C., Jin M., Wei L., Yang L., Zhou J. (2021). Identification and biological characterization of a new pathogen that causes potato scab in Gansu Province, China. Microb. Pathog..

[73] Shi D.Y., Wei B.F., Li H.P., Zeng J.Y., Wang H.L., Li Y.L. (2023). Evaluation on the control effect of four biological fungicides against verticillium wilt of Cotinus. For. Pest Dis..

[74] European and Mediterranean Plant Protection Organization (EPPO) (2014). EPPO Standard PP 1/135(4): Efficacy Evaluation of Plant Protection Products—Phytotoxicity Assessment. EPPO Bull..

