# Supplementary material for: Identification and Chemical Control of Stem Canker Pathogen of Idesia polycarpa
Source: Plants (Basel). 2025 May 5;14(9):1393. doi: 10.3390/plants14091393 (PMC12073612; doi:10.3390/plants14091393)
Supplement: Supplementary file 1 [file plants-14-01393-s001.zip › Figure S2.pdf]

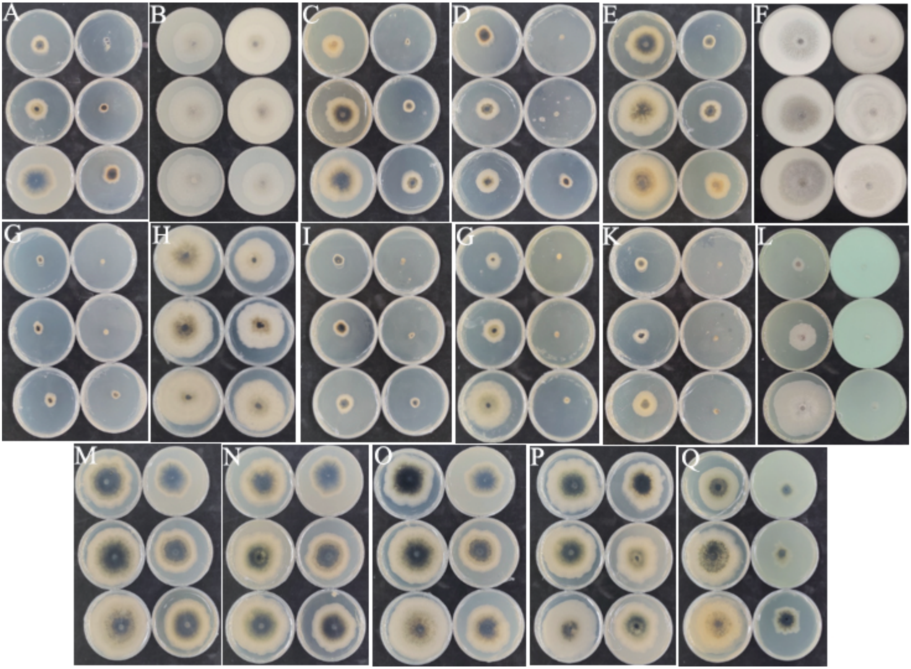

The antibacterial activity of 17 fungicides. A: 40% Thiabendazole; B: Azoxystrobin; C: 30% Metalaxyl-M-metidaxyl; D: 43% Tebuconazole; E: 20% Thiediazole copper; F: Lime sulfur; G: 20% Xinjunan acetate; H: 36% Kasugamycin-oxine-copper; I: 40% Difenoconazole; J: 6% Kasugamycin; K: 70% Thiophanate-Methyl; L: 47% Kasugamycin-copper oxychloride; M: 27% Bromothalonil; N: 33.5% Oxine-copper; O: 25% Oligosaccharins-ethylicin; P: 30% Zincthiazole; Q: 80% Bordeaux mixture.
