# Supplementary figures and images for: Identification and Chemical Control of Stem Canker Pathogen of Idesia polycarpa
Source: Plants (Basel). 2025 May 5;14(9):1393. doi: 10.3390/plants14091393 (PMC12073612; doi:10.3390/plants14091393)

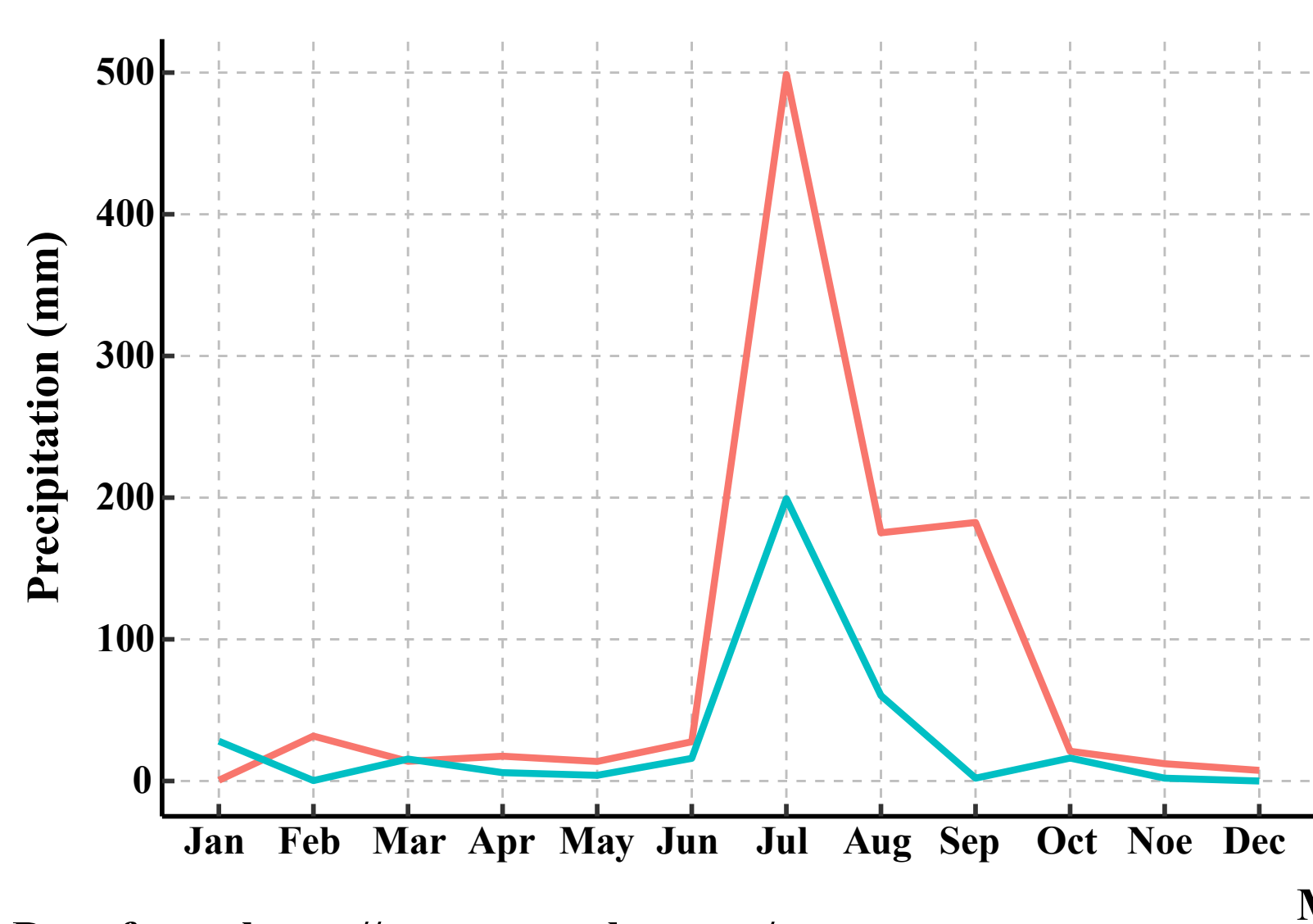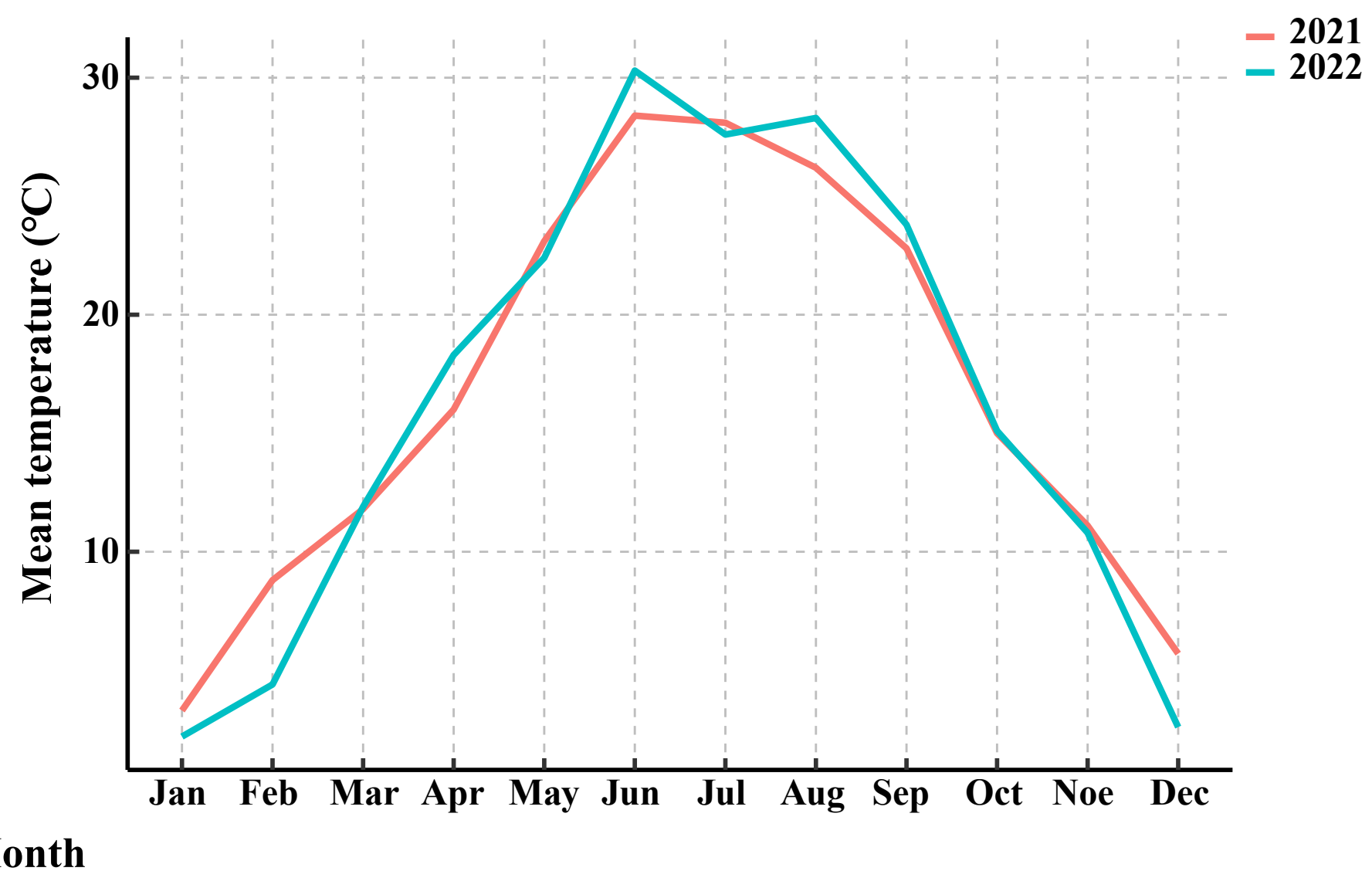

Data from: <https://www.qweather.com/>

Supplement: Supplementary file 1 [file plants-14-01393-s001.zip › Figure S1.pdf]
